# The Potential Roles of Blood–Brain Barrier and Blood–Cerebrospinal Fluid Barrier in Maintaining Brain Manganese Homeostasis

**DOI:** 10.3390/nu13061833

**Published:** 2021-05-27

**Authors:** Shannon Morgan McCabe, Ningning Zhao

**Affiliations:** Department of Nutritional Sciences, The University of Arizona, Tucson, AZ 85721, USA; morgans3@email.arizona.edu

**Keywords:** manganese, blood–brain barrier, blood–cerebrospinal fluid barrier, choroid plexus

## Abstract

Manganese (Mn) is a trace nutrient necessary for life but becomes neurotoxic at high concentrations in the brain. The brain is a “privileged” organ that is separated from systemic blood circulation mainly by two barriers. Endothelial cells within the brain form tight junctions and act as the blood–brain barrier (BBB), which physically separates circulating blood from the brain parenchyma. Between the blood and the cerebrospinal fluid (CSF) is the choroid plexus (CP), which is a tissue that acts as the blood–CSF barrier (BCB). Pharmaceuticals, proteins, and metals in the systemic circulation are unable to reach the brain and spinal cord unless transported through either of the two brain barriers. The BBB and the BCB consist of tightly connected cells that fulfill the critical role of neuroprotection and control the exchange of materials between the brain environment and blood circulation. Many recent publications provide insights into Mn transport in vivo or in cell models. In this review, we will focus on the current research regarding Mn metabolism in the brain and discuss the potential roles of the BBB and BCB in maintaining brain Mn homeostasis.

## 1. Manganese Dyshomeostasis and Neuropathological Consequences

Manganese (Mn) is essential for life as it is necessary for the normal function of several enzymes, including the antioxidant enzyme Mn superoxide dismutase (MnSOD) [1] and the neurotransmitter synthesis enzyme glutamine synthetase [2]. Since adequate Mn is easily obtained through a healthy diet, Mn deficiency is uncommon. However, Mn overload occurs more frequently and becomes a public health concern. Exposure to high levels of Mn in occupational environments such as mining, welding, and dry cell battery production can lead to manganism, which is a disorder characterized by serious and irreversible neurological symptoms similar to those seen in Parkinson’s disease. Early symptoms of manganism caused by occupational hazards include neurobehavioral changes such as impulsiveness and irritability, followed by changes in gait and difficulty with speech as the disease progresses [3]. High Mn levels in local drinking water, along with elevated Mn in blood and hair samples, reveals a correlation between higher Mn levels and decreased memory, verbal, and overall IQ scores [4]. Elevated environmental Mn exposure in children is also correlated with poorer academic achievement [5], altered performance on visual perception and memory tasks [6], and reduced Full Scale IQ [7].

In patients with mutations in Mn-transport proteins, excess Mn accumulates in the blood and brain, causing neurological symptoms. Blood Mn levels in healthy individuals is <320 nmol/L, while patients experiencing neurological symptoms of Mn overload have levels exceeding 2500 nmol/L [8,9]. Additional data from patients with inherited disorders of Mn homeostasis have been recently summarized [10]. Individuals can also receive excess Mn from environmental sources. A group of people living in an area with high Mn in drinking water (1.8–2.3 μg/mL) experienced many of the neurological symptoms related to manganism, such as tremors, gait disturbances, and memory dysfunction [11], thus highlighting the dangers of excess Mn to neurological health.

Older adults are also at risk of the neurological effects of excess Mn in the brain. Alzheimer’s disease (AD) and related dementias are a group of neurological disorders that first present as cognitive impairment in aging individuals. There is no known cause for late-onset AD, but environmental pollutants such as heavy metals are thought to be a contributor [12]. In the brain, reactive oxygen and nitrogen species (ROS/NOS) are normally produced at manageable levels during oxidative phosphorylation. MnSOD is an antioxidant enzyme that requires Mn, but excess Mn reduces its antioxidant activity. In the brain of a patient with AD, there is a decrease of MnSOD activity and increased oxidative stress [13,14]. In non-human primates, chronic Mn exposure induced amyloid-beta precursor-like protein 1 expression and increased the formation of amyloid plaques, which is one of the main neuropathological hallmarks of AD [15,16]. A recent study used a transgenic mouse model of AD and exposed subjects to additional Mn via drinking water (0.36 mg/mL) over five months [17]. At the end of the study, mice consuming Mn-treated water had more beta amyloid deposition in the cortex and hippocampus than untreated transgenic mice. This result shows that Mn consumption may contribute to the severity of AD. In another study, mice were administered daily MnCl_2_ doses of either 15 mg/kg or 60 mg/kg intraperitoneally. The study concludes that increased Mn exposure is correlated with increased amyloid-beta in the blood and decreased cognitive test scores in mice [18]. These results suggest that brain Mn dyshomeostasis may be a factor in the development of AD.

## 2. Structure of the Brain Barriers

**Figure 1:**
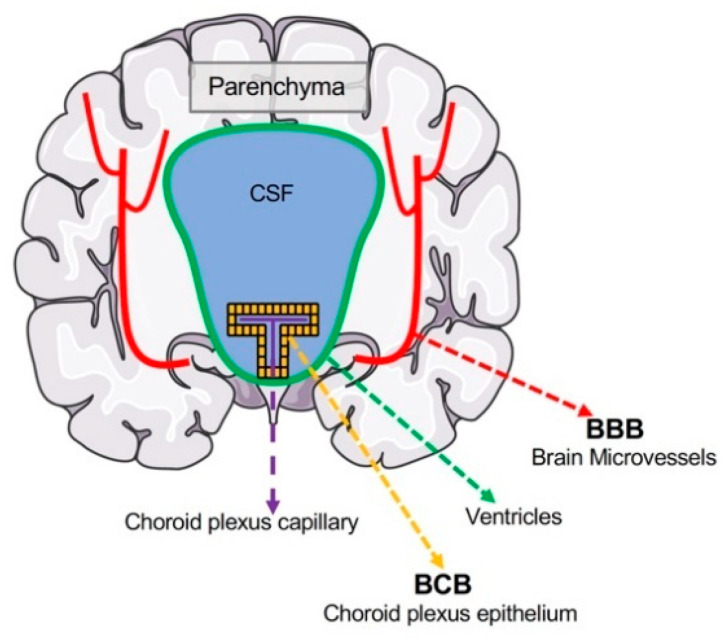
Localizations of the brain barrier interfaces. The blood–brain barrier (BBB) is localized to the microvasculature of the central nervous system and separates the lumen of cerebral blood vessels and brain parenchyma. Neurons and glia are found in the CNS parenchyma and thus protected from the periphery by the BBB. The blood–CSF barrier (BCB) is formed mainly by the choroid plexus epithelium located between choroid plexus capillaries and the CSF. Materials transported through the choroid plexus epithelium reach the CSF, where they can diffuse into the brain parenchyma.

The brain has developed physiological barriers to selectively restrict the exchange of ions and solutes between the blood and brain, allowing a tight regulation of the brain microenvironment for proper neuronal function. In order to enter the brain microenvironment, Mn from the systemic circulation has to cross either of the two strictly controlled blood–brain interfaces: the blood–brain barrier (BBB) and the blood–cerebrospinal fluid (CSF) barrier (BCB). Therefore, the BBB and BCB are the points of restriction for Mn entering the brain from systemic circulation (Figure 1). The accumulation of Mn within the brain and the export of excess Mn back into blood circulation occurs mainly across these two barriers.

The unique structures of the brain barriers provide insights into which cell types might express metal transporters. Further, cell models of the BBB and BCB may reflect the physiological structure and features of each barrier. Brain vasculature delivers oxygen and nutrients throughout the brain and shuttles toxins and unneeded materials away from the central nervous system. Unlike other organs, the exchange of molecules between the blood vessel and the brain environment is tightly regulated to prevent the infiltration of harmful pathogens, toxins, and immune factors. The BCB also restricts the movement of molecules between the blood and CSF. In essence, the brain environment beyond the blood vessel or CP epithelium is separated from general blood circulation.

### 2.1. Structure of the BBB

**Figure 2:**
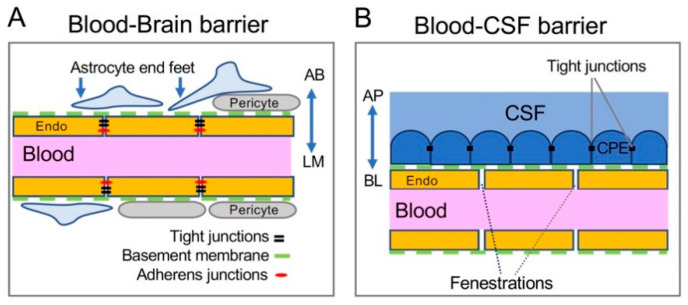
Cellular structures of the brain barriers. (**A**) The BBB is composed of endothelial cells (endo) of the brain blood vessel, and it is supported by pericytes, basement membrane proteins (green dashed line), and astrocytic end feet. The luminal (LM) side of BBB endothelial cells faces the inside of the blood vessel. It is also referred to as the apical side. The abluminal (AB) side faces the brain parenchyma and can exchange between the endothelial cell and the astrocytic end foot or brain extracellular space. It can be considered the basolateral side of BBB endothelium. (**B**) The BCB is made up of choroid plexus epithelial (CPE) cells connected to each other by tight junctions and attached to the blood vessel via basement membrane proteins (green dashed line). The apical (AP), or CSF-facing side of the CPE expresses transporters necessary for the secretion of CSF. On the basolateral (BL), or blood-facing side, CPE cells exchange materials with circulating blood, since endothelial cells in the CP lack tight junctions and permit larger molecules to diffuse.

Regulatory control of the BBB is provided by specialized barrier cells and their unique structures and junction proteins. The BBB primarily consists of three unique cell types (Figure 2A): endothelial cells of the brain blood vessels, astrocytic end feet encasing the endothelium, and pericytes that form a basement membrane between the blood vessel and astrocyte [19,20]. Endothelial cells of the BBB are polarized, with the abluminal surface toward the brain environment, and the luminal surface facing the blood vessel lumen (Figure 2A). These endothelial cells are linked by tight junctions and adherens junctions to prevent the paracellular movement of water-soluble molecules.

Tight junction proteins exist almost entirely on the interior, protoplasmic face of the endothelial cell membrane. One group of these proteins are zonula occludens-1 and -2 (ZO-1, ZO-2). Both ZO proteins are required for the formation of tight junction strands between endothelial cells in the BBB [21,22]. Junctional adhesion molecule (JAM) proteins are another group of tight junction proteins [23]. Of the three JAM proteins found in the BBB, no individuals appear to be necessary for BBB integrity [23]. However, JAM proteins are highly enriched in BBB tight junctions and are responsible for the apical–basal polarity of endothelial cells, and therefore contribute to BBB formation [24]. Other components of BBB tight junctions are the claudins and occludin. Claudin-5 is the only protein of its family that appears to be localized to the BBB and contributes to barrier integrity [23]. It is also particularly enriched in the brain endothelium over other peripheral blood vessels, indicating the importance of claudin-5 in the formation of the BBB. Similar to claudin, occludin is an endothelial transmembrane protein. Mice with occludin deficiency had increased calcium precipitation in the brain despite normal serum calcium concentration, suggesting that occludin may be necessary for BBB tight junction integrity [25].

Adherens junctions between endothelial cells of the BBB help maintain the integrity of this barrier by regulating the adhesion between cells and controlling the flow of molecules between the blood and brain [26]. Adherens junctions are primarily composed of vascular endothelial cadherins, catenins, and nectins. Cadherins interact with catenins to facilitate their linkage to the actin cytoskeleton, forming the cadherin-based adhesions between the BBB endothelial cells [23], while nectins promote the establishment of endothelial apical–basal polarity and contribute to adherens junction integrity [27].

Each of these junctions exist between endothelial cells of the BBB, but other cell types are necessary for the barrier structure and function. Pericytes are found in the basement membrane of capillaries and surround the vessel. Differences in pericyte population sizes suggest that pericytes are directly involved in BBB permeability but do not alter tight junction formation [28]. Contractile smooth muscle cells fully surround arterioles to provide blood flow control [29]. Both pericytes and smooth muscle cells assist in the structural development of the brain blood vessels [23]. Surrounding the majority of the abluminal blood vessel are astrocytic end feet. Astrocytes associated with BBB endothelial cells increase the integrity of the BBB by decreasing the permeability of tight junctions [19,30]. Astrocytes function as an extensive network and interact with each other via gap junctions to coordinate ion changes [31], while their end feet are specifically responsible for the exchange of ions and molecules with the blood vessel in order to maintain ion homeostasis [32]. A basement membrane layer fills the gap between the endothelial cells and astrocytes, with pericytes and smooth muscle cells embedded within. Pericytes and endothelial cells form a 3D structure of laminins, nidogens, collagens, and heparan sulfate proteoglycans [33,34]. Communication and transport between the blood and astrocytes occurs through this matrix.

In most other tissues, blood vessels have small gaps, or fenestrations, between endothelial cells to allow larger molecules to cross from blood to tissue. The BBB is considered a physical barrier due to its lack of fenestrations [20], presence of tight junctions, and lack of permeability to large molecules [35]. Transport of hydrophilic molecules, such as glucose and metal ions, requires specific transporters to cross the endothelial membrane, while large molecules can cross via receptor-mediated endocytosis [20].

This highly selective barrier exists in all brain blood vessels, with the exception of the vessels in the meninges and those near the circumventricular organs. The pituitary and pineal glands, as well as the median eminence, paraphysis, and area postrema, possess a less restrictive barrier in order to allow signaling molecules and hormones to reach specific brain areas, without crossing the BBB into off-target areas [36].

### 2.2. Structure of the BCB

The other major brain barrier is the BCB, which is localized to the choroid plexus (CP) within the four brain ventricles (Figure 2B). CP tissues in the left and right lateral, and third ventricles are made up of epithelial cells surrounding the anterior choroidal and posterior choroidal arteries, while the fourth ventricle epithelium receives blood flow from the anterior and posterior inferior cerebellar arteries [37]. A thin endothelial basement membrane lies on the abluminal side of the blood vessel [38]. In contrast to the blood vessels forming the BBB, the capillaries of the CP are highly fenestrated and lack tight junctions to connect the endothelial cells, allowing the movement of larger molecules from the blood vessel to the CP tissue. These molecules first reach the stroma, which is a layer of fibroblastic mesenchymal-like cells that surround the CP blood vessels [37,39,40]. Leukocytes, macrophage, and dendritic cells are known to migrate to this cell layer from the blood vessel before being transported across the epithelial layer into the brain [40,41,42].

The outermost layer consists of polarized CP epithelial cells that are connected to the basement membrane and stromal layer on their basolateral side, allowing the cells to interact with systemic blood circulation while the apical, CSF-facing side is responsible for producing CSF and exchanging materials with the ventricles [40,43]. The presence of microvilli on the apical brush border of the epithelial cells increases the surface area and facilitates the transport of molecules into the ventricle [44].

CP epithelial cells are connected by tight junctions, thus restricting free passage of large or hydrophilic molecules into and out of the brain. While knowledge of CP tight junction proteins is less complete than that of the BBB, it is currently understood that many tight junction proteins of the BBB are also expressed in CP epithelial cells. The decreased occludin level induces epithelial permeability to larger molecules, suggesting that occludin may be a necessary component for the formation of CP tight junctions to block the transfer of large molecules across this barrier [45]. The expression of various claudins in the CP epithelium may be specific to developmental stages and species [46,47]; however, consistent reports of human and murine tight junctions show that claudin-1, -2, and -3 can be detected in CP epithelial tight junctions [48,49]. As in the BBB, the epithelial cells of the BCB express intracellular accessory protein ZO-1 [50] that seems to be required for tight junction integrity, since decreases in ZO-1 expression by inflammation cause an increase in the BCB permeability [51]. Lining all other surfaces of the ventricular walls are ependymal cells—cuboidal epithelial cells that lack tight junctions and are permeable to macromolecules [52]. Since the only structure between the CSF and brain parenchyma is this permeable ependymal cell barrier, molecules in the CSF could enter brain parenchyma by diffusion. Thus, the BCB function is carried out mainly by the single layer of CP epithelial cells and the tight junctions that link them [53,54].

## 3. Mn Homeostasis at the Brain Barriers: Evidence of Involved Metal Transporters

### 3.1. Potential Roles of Iron Transport Pathway Proteins in Mediating Mn Delivery at the Brain Barriers

#### 3.1.1. Transferrin (Tf)

Many divalent metals share the same set of transporters. Studies of iron transport and absorption led to the first understandings of Mn homeostasis, particularly in studies of transferrin/transferrin receptor 1 (Tf/TfR1) and divalent metal transporter-1 (DMT1). The Tf cycle is the primary pathway for cells to take up iron. In this pathway, the circulating Tf carries Fe^3+^, followed by binding to the cell surface TfR1 and subsequent invagination into intracellular vesicles. The acidic pH inside the vesicles causes the release of Fe^3+^ from Tf. Then, Fe^3+^ is reduced to Fe^2+^ and transported into the cytoplasm via DMT1 [55,56]. In addition to Fe^3+^, Tf can bind to trivalent Mn (Mn^3+^) [57,58] because Mn^3+^ is very similar in structure to Fe^3+^ [59]. Moreover, Mn and Fe accumulate in many of the same brain areas during overload conditions [60]. Therefore, the transport of Mn is presumably tied to the proteins involved in iron transport, including Tf. However, it has been shown that mice with Tf deficiency had similar levels of Mn in the brain compared to the wild-type animals, indicating that Tf is not necessary for the delivery of Mn to the brain [58].

#### 3.1.2. DMT1

As mentioned above, DMT1 is a metal transporter that mediates the efflux of divalent metals from a vesicle to the cytoplasm. DMT1 functions optimally at pH 5.5, but its functionality in cells at pH 7.4 has also been observed [61]. DMT1 can localize to the plasma membranes of enterocytes or hepatocytes in high- or low-Fe conditions, respectively; whereas in regular dietary conditions, DMT1 remained primarily in the cytoplasm [62]. While DMT1 can adapt to changing substrate availability and subcellularly localize accordingly, its expression in the brain barriers is low. DMT1 mRNA expression is very low in isolated rat brain capillaries [63] and brain endothelial cells in culture [55]. In addition, protein expression is not detectable in brain endothelial cells of adult or early postnatal mice [64]. In a developmental study carried out in rats, the expression of DMT1 was detected by immunohistochemistry in the choroid plexus during early postnatal days, with increased expression at postnatal day 15. DMT1 was detected when staining cerebral blood vessels, but it aligned very closely with astrocyte localization; therefore, the transporter could be present on either the endothelial or glial cells [65]. In contrast, adult rats appear to express DMT1 protein in the CP epithelial cells, but not in microvascular endothelial cells [66], and the expression of DMT1 in CP epithelial cells was observed primarily in the cytoplasm.

A study of brain microvascular endothelial cells of human origin (hBMVEC) provides an analysis of time-dependent uptake of ^54^Mn^2+^ that increased in the presence of a clathrin-dependent endocytosis inhibitor [67], demonstrating that the receptor-mediated endocytosis in the Tf/TfR1 and DMT1 pathway is not involved in Mn transport in this BBB cell model. Moreover, in Belgrade rats that lack functional DMT1, brain Mn levels remained normal in the olfactory bulb, cortex, striatum, hippocampus, and cerebellum, while Fe levels decreased in all brain areas tested [68], suggesting that DMT1 is not required for Mn delivery into the brain.

### 3.2. ZIP- and ZnT-Family Transporters

#### 3.2.1. ZIP8 and ZIP14

ZIP14 and ZIP8 are two recently identified members of the Zrt- and Irt-like protein family of metal transporters. Both proteins have been investigated for their roles in brain Mn homeostasis [10,69,70,71,72].

In polarized HIBCPP cells, a cell model for the BCB epithelium, ZIP14, was enriched on the basolateral membrane, while ZIP8 was enriched on the apical membrane [73]. The knockdown of ZIP14 or ZIP8 using siRNA-mediated technology led to a decrease in ^54^Mn accumulation in HIBCPP cells, although the decrease in ^54^Mn accumulation was much greater with ZIP14 knockdown [73]. These results suggest that both ZIP14 and ZIP8 are involved in Mn uptake in this CP epithelial cell model, which is consistent with previous studies on epithelial cell models of intestine [70], lung [74], and liver [75].

In human primary brain microvessel endothelial cells (hBMVEC), a cell model for the BBB endothelium, the expression of both ZIP14 and ZIP8 were identified [67]. The uptake of ^54^Mn was dependent on both ZIP8 or ZIP14, with significantly decreased ^54^Mn accumulation when one or both proteins were knocked down. In contrast to the expression pattern observed in HIBCPP cells, ZIP14 and ZIP8 were localized to both sides of the polarized hBMVEC cells, where both proteins seem to be involved in apical-to-basolateral and basolateral-to-apical transport of Mn. Flux in the basolateral-to-apical direction was more prominent, modeling the movement of Mn from the brain to the blood through the BBB.

These studies using cell models of the BCB and BBB may provide insights into how Mn is transported within these two barriers. By identifying the polarized localization of ZIP14 and ZIP8 in CP-derived cells, we can begin to understand how Mn is transported at the BCB. Apical ZIP8 expression in HIBCPP cells suggests that Mn could be transported from the CSF into the epithelial cells to facilitate apical-to-basolateral movement of Mn out of the brain. In the same way, basolateral ZIP14 expression in CP-derived epithelial cells indicates that ZIP14 could be involved in the blood-to-brain movement of Mn via import of Mn from the blood to the epithelial cell of the BCB. The uptake experiments in hBMVEC endothelial cells suggest that both ZIP8 and ZIP14 play a significant role in Mn uptake into endothelial cells of the BBB. The basolateral-to-apical flux of Mn in these cells translates to a brain-to-blood movement of Mn in vivo. Thus, the BBB could have a considerable role in Mn clearance from the brain, dependent on coordinated transport by ZIP8 and ZIP14. A future study in the CP-derived HIBCPP cells could be useful to indicate the direction of Mn transport in cells with polarized expression of Mn transporters.

#### 3.2.2. ZnT10

ZnT10 is a member of the Zinc Transporter family proteins. Patients with an inherited homozygous *ZnT10* mutation resulting in a non-functional ZnT10 protein exhibit high Mn levels in the blood and brain, as well as Mn toxicity-induced dystonia [76,77,78]. In cell culture studies, ZnT10 appears to be a Mn efflux transporter [79] expressed on the surfaces of enterocytes [78] and neuronal cells [80]. In both humans and mice, ZnT10 was highly expressed in the brain, liver, and intestine [78,80,81].

There is evidence showing that ZnT10 mRNA is expressed in the CP in rats [82], but there is no evidence to show that ZnT10 is expressed in the brain microvessels at either the gene or protein level. A recent study used pan-neuronal/glial *Znt10* knockout mice and detected no difference in brain Mn levels with standard dietary conditions [78]. In this study, *Znt10* knockout mice lack the protein in the vast majority of brain cells, including all neurons, astrocytes, and oligodendrocytes. As an efflux transporter, ZnT10 would likely protect neurons and glia from high Mn levels when overloaded, but a lack of increased Mn in the knockout mice indicates that these brain cells are not accumulating Mn, at least in normal conditions. Thus, a normal amount of Mn was circulating in the interstitial fluid and CSF, regardless of ZnT10 expression. This finding provides key information about Mn balance within the brain parenchyma, but the brain environment is controlled by the BCB and BBB. Therefore, Mn levels within the brain may not change without a change in transporter expression in either of the brain barriers. Interestingly, *Znt10* neuronal/glial knockout mice exposed to high dietary Mn experienced a greater increase of Mn in specific brain areas compared to exposed wild-type littermates. This result suggests that there is less Mn transported out of the brains lacking ZnT10, which could indicate that the efflux transporter is normally localized to either the brain endothelium or CP epithelium.

The expression pattern of ZnT10 in the BBB or BCB is unknown. As an efflux transporter, cell type localization of ZnT10 is necessary to understand which barrier is responsible for Mn efflux. Additionally, ZnT10 is likely polarized to either the basolateral or apical surfaces of the epithelial or endothelial cells. If polarized, its location would indicate whether ZnT10 is responsible for brain Mn accumulation or clearance. In addition, cell culture models of BBB and BCB are ideal to elucidate Mn transport mechanisms at the cellular level. Uptake and transport studies, such as those completed in previous studies of ZIP8 and ZIP14, would indicate if ZnT10 is necessary for normal Mn accumulation or efflux. Additionally, transport studies in polarized epithelial or endothelial monolayers would confirm the direction of Mn transport to which ZnT10 contributes.

### 3.3. ATP13A2

Another transporter associated with brain Mn homeostasis is ATP13A2. Mice with *Atp13a2* knockout accumulated more Mn in the brain compared to wild-type mice after intraperitoneal administration of MnCl_2_ [83]. Since brain Mn levels tend to rise when the blood levels of Mn increase, a future investigation should report blood or serum levels in order to further understand the location of accumulated Mn in *Atp13a2* knockout mice. While knockout of *Atp13a2* causes brain Mn accumulation, overexpression of ATP13A2 in HeLa cells and nematode dopamine neurons had a protective effect against high Mn exposure [84]. Taken together, ATP13A2 appears to have a role in Mn homeostasis within the brain, but it is unclear how it could act as a Mn transporter at the BBB or BCB. To date, there are no publications showing evidence of ATP13A2 in human or mouse brain endothelium or choroid plexus tissue. Future studies of ATP13A2 should identify the transporter’s tissue and membrane localization within the brain barriers to determine if the protective effect of this transporter against high Mn exposure is applicable to CP epithelial cells and brain endothelial cells. Major evidence of the metal transporters’ involvement in Mn homeostasis at the BBB and BCB is summarized in Table 1.

## 4. Brain Mn Accumulation Is Likely to Occur via the BCB

The BBB and BCB are required to maintain the normal physiological conditions of the central nervous system. These two barriers have distinct but overlapping roles in the exchange of material from the blood to brain, as demonstrated by the difference in transporter expression and transport activity in each barrier. For example, both the CP and the brain endothelium express glucose transporters that deliver energy to the brain via facilitated diffusion [85]. Glucose is transported into the brain through both barriers, although it is estimated that the BCB imports only about 1/100th of the glucose that the BBB transports [86,87]. This pattern of uneven transport may also be applicable to Mn distribution into the brain.

A cell culture study of Mn transport across porcine BCB and BBB models indicated that the BCB is likely the primary route for brain Mn uptake. First, uptake studies indicated that CP epithelial cells accumulate nearly three times more Mn than endothelial cells when exposed to the same amount of MnCl_2_ in media. Second, in a Transwell model of polarized cells with MnCl_2_ added to each side, it was found that epithelial cells accumulated significantly more Mn in the apical chamber, suggesting that CP epithelial cells predominantly transport Mn in the basolateral-to-apical direction. In the BBB model, endothelial cells did not accumulate more Mn in one chamber than the other, indicating that the BBB transports Mn in both directions equally [88]. A future study might compare these findings with lower Mn concentrations that could be more relevant to physiological or Mn-overload conditions. Nevertheless, the results from this study provide valuable information about the activity of Mn transport across the brain barriers. Importantly, if these results reflect the in vivo behavior of the BCB, increased blood Mn would cause higher basolateral-to-apical Mn transport through the CP epithelium.

In vivo studies also provide evidence for the primary role of the BCB in brain Mn uptake. Brain Mn-mapping studies carried out in animals using peripheral Mn^2+^ administration followed by enhanced magnetic resonance imaging suggest that the entry of Mn into the CNS occurs predominantly through BCB. First, in mice, 2 h after intraperitoneal MnCl_2_ injection, the Mn signal was first enhanced in the CSF-containing ventricles. This ventricular signal cleared over the next 24 h accompanied by a gradual increase of parenchymal Mn intensity. A close examination of different brain regions revealed that the Mn signal was highest in areas immediately adjacent to the CSF-containing ventricles, while the signal intensity steadily decreased with increasing distance from the ventricles [89]. Second, in rats, within 5 min of MnCl_2_ injection through the tail vein, the Mn signal was first enhanced in the choroid plexus. At 10 min, the signal diffused to the entire CSF-containing ventricles, and by 100 min post injection, the Mn signal spread into the periventricular tissues that are in contact with the CSF [90]. Third, in marmosets, 1.5 h after the start of MnCl_2_ infusion through the tail vein, the Mn signal was initially enhanced in the CP, and at the 2.5 h time point, the signal was detected in the parenchyma surrounding the ventricles. In contrast, throughout the entire 6 h infusion course, no Mn signal was detected in brain regions that are not adjacent to the ventricles [91].

These findings in cell models and animals suggest that a main route for Mn uptake into the brain is from the CP, through the CSF, and then to the brain parenchyma. The cell culture studies suggest that the BBB has a role in Mn transport, but it does not cause the accumulation of Mn in the brain from the blood. Meanwhile, the BCB preferentially transports Mn from the blood into the brain, potentially contributing to brain Mn overload. Since the brain endothelial cells appear to transport Mn in both directions, and CP epithelial cells transport more Mn into the brain than into the blood, future studies in cell models or in vivo could investigate transporters involved in unidirectional or bidirectional transport of manganese across endothelial or epithelial cells.

## 5. Future Directions

To further understand Mn homeostasis across the brain barriers, more in vitro models of the BBB and BCB that reflect the barrier qualities of each cell layer need to be developed. Cells modeling the BBB or BCB must polarize, form tight junctions, and prevent the diffusion of large or hydrophilic molecules. Such models are necessary to identify the efflux and influx of Mn through the cells of the brain barriers at different Mn concentrations. For example, Mn accumulation studies in animals indicated that Mn crosses the choroid plexus and quickly travels into the CSF, suggesting a major role for the BCB in Mn uptake. Cellular transport experiments in CP epithelial cells could distinguish how Mn is transported across the basolateral and apical membranes and would bolster the conclusion that the BCB is the primary site of Mn absorption into the brain. Due to the growing knowledge of Mn metabolism, modulators of Mn transport may be developed in the near future, but no such technology exists at this time. Fundamental research of brain Mn homeostasis may eventually facilitate the development of methods to control the balance of metals in the brain to limit the negative effects of excess Mn.

In vivo research is a necessary step to establish Mn transport mechanisms, but there are a few limitations of animal research in this area. To study Mn transport, there is the difficulty of identifying where Mn is concentrated within the brain parenchyma. In Mn overload conditions, it is unknown whether Mn accumulates in neurons and glia of specific brain regions or within the interstitial fluid and CSF. Most publications report brain Mn levels as the level of Mn in the whole brain homogenate, making it difficult to distinguish between Mn accumulating in brain cells or CSF and interstitial fluid. Future studies are needed to understand this important distinction. To function and fire quickly, neurons rely on steep ionic gradients between their intracellular environment and the surrounding interstitial fluid. Since the concentrations of Ca^2+^, Na^+^, K^+^, and Cl^−^ would be vastly different when sampling either neurons or the extracellular fluid, we could logically understand that levels of other charged ions such as Mn^2+^ would be different within the neuron or out in the interstitial fluid. Additionally, astrocytes are known to release and take up ions and nutrients, while the brain has changing demands for these materials. Astrocytes may sequester Mn intracellularly or release it back into the interstitial fluid, leaving the total brain Mn concentration unchanged. When using a whole brain homogenate to measure metal levels, the extracellular environment around the BCB cannot be sampled separately. To accurately reflect the Mn concentrations on each side of the BCB, Mn levels in both CSF and blood can be measured. In addition, knowledge of Mn accumulation in separate brain compartments, as well as improved understanding of transporter expression in human and animal tissues, will help make significant advances in the field of Mn homeostasis within the brain barriers.

## Figures and Tables

**Table 1:** Evidence of metal transporters involved in Mn homeostasis at the brain barriers.

Protein	Experimental Model	Major Results and Conclusions	Reference
Transferrin (Tf)	Hypotransferrinemic (Hpx) mice as a model for Tf deficiency	▪ Hpx mice had normal brain Mn accumulation. ▪ Suggests that Tf is not required for brain Mn loading.	[58]
DMT1	Human brain endothelial cells (hBMVEC) as a model for the BBB	▪ Increased Mn uptake despite inhibition of clathrin-mediated endocytosis. ▪ Suggests that DMT1 and Tf/TfR1 pathway is not necessary for Mn uptake in brain endothelial cells.	[67]
	Belgrade rats as a model for DMT1 deficiency	▪ Belgrade rats have normal brain Mn levels. ▪ Indicates that DMT1 is not necessary for brain Mn accumulation.	[68]
ZIP8	Choroid plexus epithelial cells (HIBCPP) as a model for the BCB	▪ ZIP8 knockdown reduces Mn uptake. ▪ ZIP8 is primarily localized to the apical membrane ▪ Suggests that ZIP8 may mediate apical Mn uptake into CP epithelial cells.	[73]
	hBMVEC cell model of BBB	▪ ZIP8 is expressed on both apical and basolateral membrane. ▪ ZIP8 is involved in both apical-to-basolateral and basolateral-to-apical Mn transport.	[67]
ZIP14	HIBCPP cell model of BCB	▪ ZIP14 knockdown reduces Mn uptake. ▪ ZIP14 is expressed on the basolateral membrane. ▪ Suggests that ZIP14 may mediate basolateral Mn transport into CP epithelial cells.	[73]
	hBMVEC cell model of BBB	▪ ZIP14 is expressed at both apical and basolateral membrane. ▪ ZIP14 is involved in both apical-to-basolateral and basolateral-to-apical Mn transport.	[67]
ZnT10	Pan-neuronal/glial *Znt10* knockout (KO) mice as a model for brain ZnT10 deficiency	▪ Pan-neuronal/glial Znt10 KO mice have increased Mn accumulation in certain brain areas under Mn overload conditions induced by subcutaneous Mn injection. ▪ Suggests reduced Mn efflux from the brain with ZnT10 deficiency when body Mn levels increase.	[78]
ATP13A2	*Atp13a2^−/−^* mice as a model for ATP13A2 deficiency	▪ *Atp13a2^−/−^* mice accumulate more Mn in the brain compared to the control mice after intraperitoneal administration of MnCl_2_.	[83]
	HeLa cells and *C. elegans* with ATP13A2 overexpression	▪ Overexpression of ATP13A2 protects HeLa cells from Mn-induced cytotoxicity. ▪ *C. elegans* overexpressing ATP13A2 in dopamine neurons are more resistant to Mn-induced neurotoxicity. ▪ Suggests that ATP13A2 may have a role in maintaining brain Mn homeostasis.	[84]
